# RETRACTION: Haemoglobin Changes Before and After Packed Red Blood Cells Transfusion in Burn Patients: A Retrospective Cross‐Sectional Study

**DOI:** 10.1111/iwj.70237

**Published:** 2025-02-25

## Abstract

**RETRACTION:** ParviziA. , HaddadiS. , RoshanZ. A. , and KafashP. , “Haemoglobin Changes Before and After Packed Red Blood Cells Transfusion in Burn Patients: A Retrospective Cross‐Sectional Study,” International Wound Journal 20, no. 6 (2023): 2269–2275, 10.1111/iwj.14108.36752214 PMC10333046

The above article, published online on 08 February 2023, in Wiley Online Library (http://onlinelibrary.wiley.com/), has been retracted by agreement between the journal Editor in Chief, Professor Keith Harding; and John Wiley & Sons Ltd. A third party reported to the journal that they had found evidence of excessive self‐citations in the reference list of this article. The publisher did not confirm the evidence of excessive self‐citations. Upon further investigation, the publisher also concluded that this article was accepted solely on the basis of a compromised peer review process. In addition, the article does not include any information about the informed consent procedure for the study. In view of the clear evidence of compromised peer review, the parties agreed that the paper must be retracted. The authors disagree with the retraction.

**RETRACTION:** A. Parvizi , S. Haddadi , Z. A. Roshan , and P. Kafash , “Haemoglobin Changes Before and After Packed Red Blood Cells Transfusion in Burn Patients: A Retrospective Cross‐Sectional Study,” International Wound Journal 20, no. 6 (2023): 2269–2275, 10.1111/iwj.14108.36752214 PMC10333046

The above article, published online on 08 February 2023, in Wiley Online Library (http://onlinelibrary.wiley.com/), has been retracted by agreement between the journal Editor in Chief, Professor Keith Harding; and John Wiley & Sons Ltd. A third party reported to the journal that they had found evidence of excessive self‐citations in the reference list of this article. The publisher did not confirm the evidence of excessive self‐citations. Upon further investigation, the publisher also concluded that this article was accepted solely on the basis of a compromised peer review process. In addition, the article does not include any information about the informed consent procedure for the study. In view of the clear evidence of compromised peer review, the parties agreed that the paper must be retracted. The authors disagree with the retraction.

